# Impact of temperature variability on childhood allergic rhinitis in a subtropical city of China

**DOI:** 10.1186/s12889-020-09531-6

**Published:** 2020-09-17

**Authors:** Xu Wang, Jian Cheng, Li Ling, Hong Su, Desheng Zhao, Hong Ni

**Affiliations:** 1 Department of Science and Education, Children’s Hospital of Anhui Medical University (Anhui Provincial Children’s Hospital), No.39, Wangjiang East Road, Hefei, 230032 Anhui Province China; 2 Department of Epidemiology and Health Statistics, School of Public Health, Anhui Medical University, Hefei, Anhui China; 3 School of Public Health and Social Work, Institute of Health and Biomedical Innovation, Queensland University of Technology, Brisbane, Queensland Australia; 4 Department of Medical Affairs, The First Affiliated Hospital of USTC, Hefei, Anhui China

**Keywords:** Temperature variability, Childhood, Allergic rhinitis, Subtropical city

## Abstract

**Background:**

Many studies have shown an association of childhood respiratory diseases with short-term temperature variability such as diurnal temperature range (DTR) and temperature change between two neighboring days (TCN). However, the impact of temperature variability on allergic rhinitis (AR) has not been investigated so far. This study sought to evaluate the short-term effect of temperature variability (i.e., TCN and DTR) on AR, as well as to identify vulnerable subpopulations.

**Method:**

We collected daily data on emergency room visits and outpatients for AR and weather variables in Hefei, China during 2014–2016. A distributed lag non-linear model that controlled for long-term trend and seasonality, mean temperature, relative humidity, day of week was used to fit the associations of AR with DTR and TCN. Stratified analyses by age, sex and occupation were also performed.

**Results:**

During the study period, there were a total of 53,538 cases and the average values of DTR and TCN were 8.4 °C (range: 1.0 °C to 21.2 °C) and 0 °C (range: − 12.2 °C to 5.9 °C), respectively. While we did not observe an adverse effect of DTR on AR, TCN was significantly associated with increased risk of AR. Specifically, a large temperature drop between two adjacent days (3.8 °C, 5th percentile of TCN) has a delayed and short-lasting effect on AR, with the estimated relative risk of 1.02 (95% confidence interval: 1.01 to 1.04) at lag 12. Moreover, boys and children older than 15 years seemed to be more vulnerable to the effect of TCN.

**Conclusions:**

This study provided evidence of an adverse effect of large temperature drops between two adjacent days on childhood AR. Attention paid to boys and older children may help prevent AR attacks.

## Background

Allergic rhinitis (AR) is an inflammatory disease of the nasal mucosa, affecting more than 600 million people worldwide, especially in children [1, 2]. In recent decades, the prevalence of AR in many countries has increased markedly [3]. Clinical symptoms of AR consist of nasal itching, sneezing, watery nasal discharge, rhinorrhea, and blocked nose [4]. Although most cases show mild and self-limiting illness, its large prevalence causes a considerable burden on both rhinitis sufferers and society, and negatively impacts the quality of life (QOL) for a large number of individuals [5].

Epidemiological studies have revealed that the prevalence of childhood AR has a general upward trend in the past 7 years, ranging from 2.2 to 14.6% among children at the age of 6–7 years, and from 4.5 to 45.5% among adolescents aged 13–14 years all over the world [6]. Specifically, the prevalence of self-reported AR was 30.0% in Beijing and 31.1% in Urumqi, respectively [7]. In Thailand, AR prevalence increased from 37.9% in the year 1995 to 50.6% in the year 2001 [8]. The severity of the rhinitis was found to have an effect on QOL, sleep, daily activities, and study performance [9], and childhood AR was significantly associated with an increased risk of incident asthma in preadolescence, adolescence, or adult life [10].

Climate change has been widely recognized as the biggest health threat in the twenty-first century, and temperature play an important role in affecting human health, including cardiovascular and respiratory diseases [11]. In recent years, there have been increasing studies assessing the association between meteorological factors and AR [12–14]. These studies consistently suggest that cold weather is an important risk factor of AR. Nevertheless, the impact of unstable weather such as diurnal temperature range (DTR) and temperature change between neighboring days (TCN) has not yet been examined in previous studies. There is some evidence that unstable weather can influence the concentration of allergens (e.g. pollen, fungal spore, dust mites) that are triggers of AR occurrence [15]. Moreover, sudden temperature change had adverse effects on respiratory and immune system such as decreased effectiveness of respiratory defenses and more inflammatory nasal responses [15], which will increase the likelihood of respiratory disease occurrence among children, as supported by previous studies demonstrating an association between temperature variability and childhood asthma and acute bronchitis [16, 17]. It is thus possible that unstable weather, i.e., DTR and TCN, is a underlying risk factor of childhood AR occurrence.

To verify our hypothesis, we collected time-series data of daily childhood AR patients and daily weather records to investigate the relationship between childhood AR occurrence and temperature variability including DTR and TCN. Stratified analyses by age, sex and occupation were also conducted to identify potential vulnerable subgroups.

## Methods

### Data collection

Hefei is the capital city of Anhui province and is located in the east central of China (31° 52′ N, 117° 17′ E). It has a population density of 688 persons per km^2^ (in 2016: population = 7,869,000 persons; land size = 11,445 km2). It has a typical temperate climate, with the annual average temperature and precipitation being 15.7 °C and 3.0 mm, respectively.

Daily AR outpatients and emergency room visits from January 1st, 2014 to December 31st, 2016 were obtained from Anhui Provincial Children’s Hospital and the diagnosis of AR patients was based on the International Classification of Diseases 10th version (ICD10, J30.1-J30.4). Anhui Provincial Children’s Hospital is the biggest and only specialist children’s hospital in Anhui province, which is located at the center of the Hefei city, with advanced facilities, more than 1200 beds and over 1700 staff. This hospital has a well-managed electronic medical record system, which can provide daily hospitalization data including name, sex, age, address, principal diagnosis and treatment date.

In addition, we also obtained data for the same period on daily maximum temperature, mean temperature, minimum temperature, relative humidity, rainfall and wind velocity from Hefei Bureau of Meteorology. In line with previous studies [15, 16], DTR was calculated as the difference between daily maximum and minimum temperatures, and TCN as the difference of mean temperature between two adjacent days.

### Data analysis

A Poisson generalized linear regression model combined with a distributed lag non-linear model (DLNM) was used to examine the relationship between TCN (or DTR) and AR occurrence. DLNM has been widely applied in studies to quantify the effects of temperature and air pollution on mortality.

We controlled for seasonality and long-term trend using a natural cubic spline with 7 df per year for time. The day of week was also included as an indicator in our analysis. Mean temperature and relative humidity were controlled in the model by using a natural cubic spline with three degrees of freedom (*df*). In all cases, the Akaike Information Criterion (AIC) and an analysis of residuals were used to evaluate the choice of *df*.

Based on the results of exploratory analyses that TCN and DTR may have a delayed effect on human health, we considered a lag (delayed effects) of up to 20 days when analyzing the influence of DTR and TCN on children AR occurrence. We also examined TCN effect on childhood AR at lag 5. To examine which groups are most likely to be affected by TCN, we stratified the regression analyses by sex (male and female), age group (0–4 years, 5–14 years and ≥ 15 years) and occupation (students, scattered children, and nursery children).

All statistical analyses were conducted using the R statistical software (version 2.15) with the ‘dlnm’ package used to fit the regression model. Sensitivity analyses were performed by varying the *df* for time, mean temperature, relative humidity, respectively.

## Results

During the study period, there were a total of 53,538 AR cases. Table 1 shows the summary statistics for AR and weather variables. Every day, there was an average of 48.8 (SD: 18.7) AR cases, among which, 67.55% were boys and 82.92% were children aged between 5 and 14 years.

**Table 1:** Summary statistics for daily weather variables, and AR cases in Hefei, China, 2014–2016

Weather and AR cases (total, sex, age, and occupation)	Sum	Mean ± SD	Frequency distribution				
			Min	P(25)	P(50)	P(75)	Max
Mean temperature (°C)	/	16.8 ± 8.9	−5.9	8.8	18.0	24.4	33.7
Relative humidity (%)	/	75.8 ± 13.2	33.0	68.0	76.0	85.0	100.0
Rainfall (mm)	/	3.6 ± 10.6	0.0	0.0	0.0	0.9	129.3
Wind velocity (m/s)	/	1.9 ± 0.8	0.3	1.4	1.8	2.3	5.9
DTR (°C)	/	8.4 ± 3.9	1.0	5.3	8.2	11.2	21.2
TCN (°C)	/	0.0 ± 2.2	−12.2	−1.1	0.2	1.4	5.9
Total	53,538	48.8 ± 18.7	0	36	46	58.8	116
Male	36,163	33.0 ± 13.5	0	23	30	41	85
Female	17,375	15.9 ± 6.6	0	11	15	20	40
0 ~ 4 years	7500	6.8 ± 6.0	0	2	5	10	36
5 ~ 14 years	44,395	40.5 ± 16.9	0	29	38	50	105
≥15 years	1643	1.5 ± 1.8	0	0	1	2	12
Nursery children	16,481	15.0 ± 6.2	0	11	14	18	43
Scattered children	19,184	17.5 ± 7.2	0	12	17	22	47
Students	17,873	16.3 ± 10.8	0	8	13	24	61

**Fig. 1:**
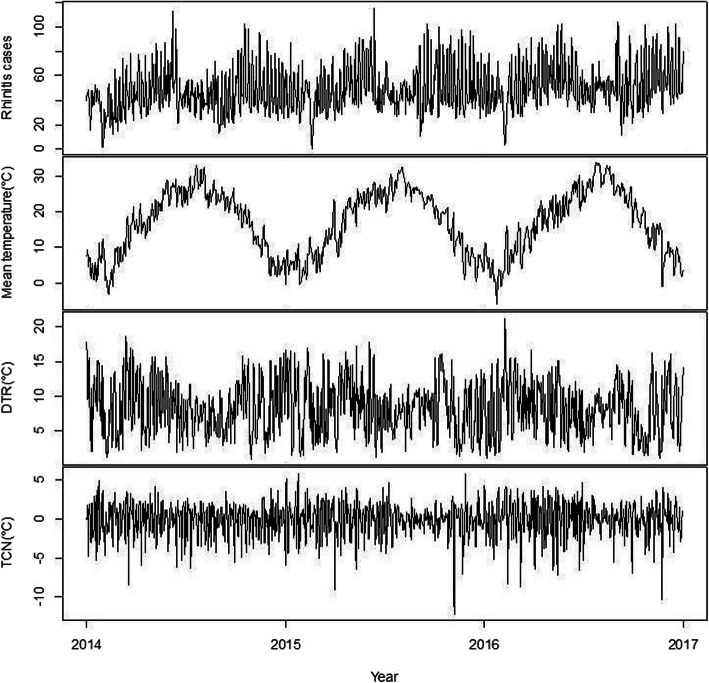
The time-series distribution of daily AR cases, daily mean temperature, DTR and TCN from 2014 to 2016 in Hefei, China

Figure 1 shows the time-series distribution of total AR cases and temperature variables (i.e., daily mean temperature, DTR, and TCN) between 2014 and 2016 in Hefei, China, suggesting an obvious seasonality in these variables.

**Fig. 2:**
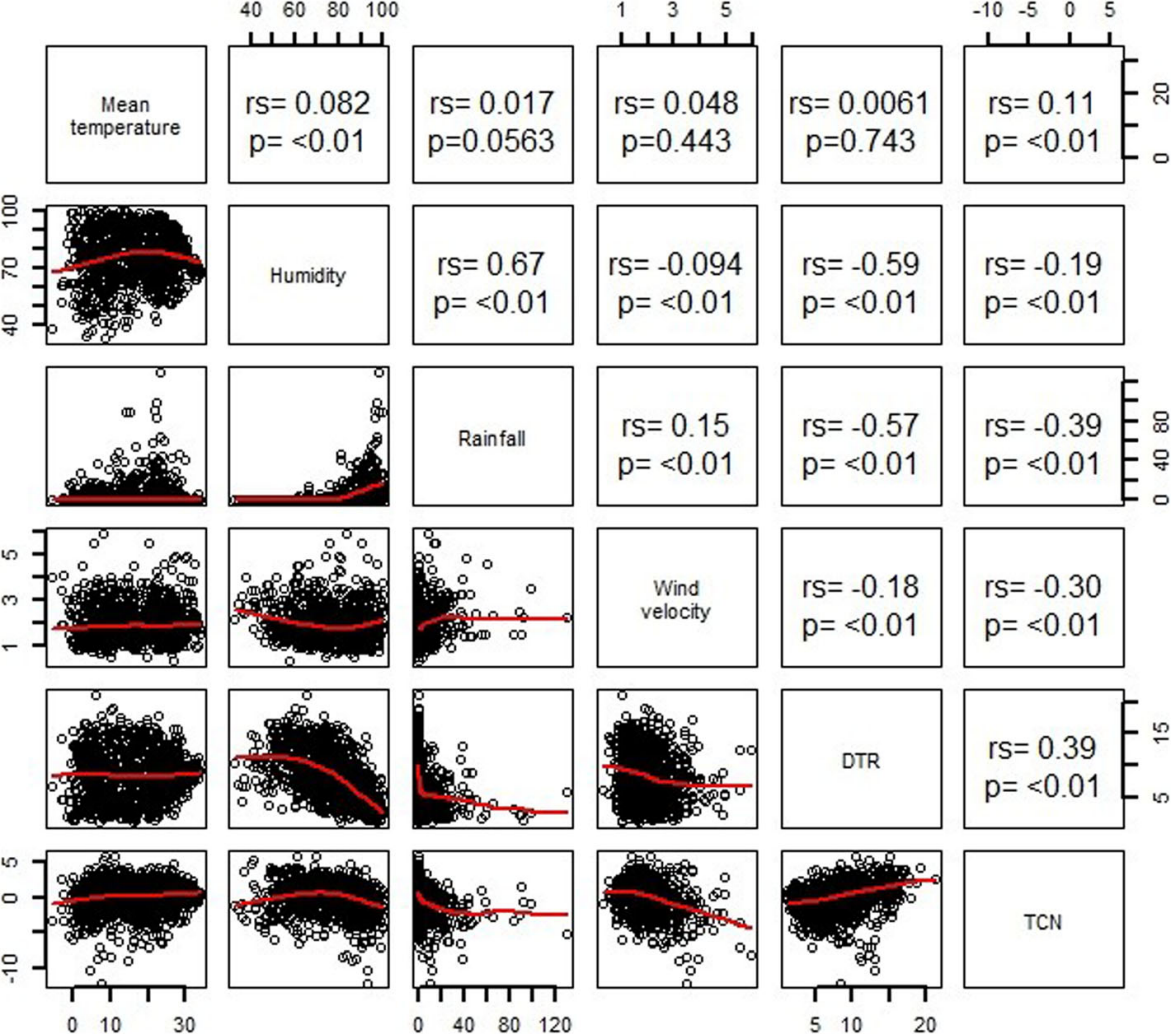
Spearman’s correlation coefficients between TCN, DTR and daily weather conditions in Hefei, China, from January 1st 2014– December 31st 2016

Figure 2 depicts the spearman correlations between weather variables. DTR was negatively correlated with humidity (rs = − 0.59), rainfall (rs = − 0.57) and wind velocity (rs = − 0.18). Similarly, TCN was negatively correlated with humidity (rs = − 0.19), rainfall (rs = − 0.39) and wind velocity (rs = − 0.30). There was statistically positive correlation between TCN and mean temperature (rs = 0.11), while correlation between DTR and mean temperature was not statistical significant.

Table 2 shows the estimated relative risk (RR) of AR at different lag days. TCN (3.8 °C, 5th percentile vs 0.2 °C, 50th percentile) had the greatest effect at lag 12. A 3.8 °C decrease in TCN was associated with an increase of 2% (95% confidence interval: 1–4%) in the daily number of AR cases in all children. We also found that subgroups including boys, children older than 15 years, and schoolchildren were more vulnerable to TCN than their counterparts.

**Table 2:** Relative risk of rhinitis admissions associated with 3.8 °C (5th percentile) decrease in temperature change between two adjacent days (Reference = 0.2 °C, 50th percentile)

AR cases (total, sex, age, and occupation)	Lag0	Lag4	Lag5	Lag8	Lag12	Lag14	Lag17	Lag20
Total	0.99 (0.98,1.01)	1.01 (0.99,1.02)	1.01 (1.00,1.03)	1.02 (1.00,1.04)	1.02 (1.01,1.04)	1.02 (1.00,1.04)	1.02 (1.00,1.04)	1.01 (1.00,1.03)
Male	1.00 (0.97,1.02)	1.01 (0.99,1.03)	1.01 (0.99,1.03)	1.02 (1.00,1.04)	1.03 (1.00,1.05)	1.03 (1.00,1.05)	1.02 (1.00–1.04)	1.02 (1.00,1.04)
Female	0.99 (0.96,1.03)	1.01 (0.98,1.03)	1.01 (0.98,1.04)	1.02 (0.98,1.05)	1.02 (0.99,1.05)	1.02 (0.99,1.05)	1.02 (0.99,1.04)	1.01 (0.97,1.04)
0–4 years	1.01 (0.96,1.06)	1.03 (0.99,1.07)	1.03 (0.99,1.07)	1.04 (0.98,1.09)	1.03 (0.98,1.09)	1.02 (0.98,1.07)	1.01 (0.97,1.05)	0.99 (0.94,1.04)
5–14 years	0.99 (0.97,1.01)	1.00 (0.98,1.02)	1.00 (0.99,1.02)	1.01 (0.99,1.03)	1.01 (0.99,1.04)	1.02 (1.00,1.03)	1.01 (1.00,1.03)	1.01 (0.99,1.03)
≧15 years	1.10 (0.98,1.23)	1.13 (1.03,1.23)	1.13 (1.03,1.24)	1.15 (1.03,1.28)	1.18 (1.06,1.31)	1.19 (1.08,1.32)	1.22 (1.12,1.33)	1.24 (1.11,1.38)
Nursery children	0.99 (0.95,1.02)	1.00 (0.97,1.02)	1.00 (0.97,1.03)	1.01 (0.97,1.04)	1.01 (0.98,1.05)	1.01 (0.98,1.04)	1.01 (0.98,1.04)	1.01 (0.98,1.05)
Scattered children	1.00 (0.97,1.03)	1.00 (0.98,1.03)	1.00 (0.98,1.03)	1.01 (0.98,1.04)	1.00 (0.97,1.04)	1.00 (0.97,1.03)	0.99 (0.97,1.02)	0.98 (0.95,1.02)
Students	1.00 (0.96,1.03)	1.02 (0.99,1.05)	1.02 (1.00,1.05)	1.04 (1.01,1.07)	1.05 (1.02,1.09)	1.05 (1.02,1.09)	1.06 (1.03,1.09)	1.06 (1.02,1.09)

**Fig. 3:**
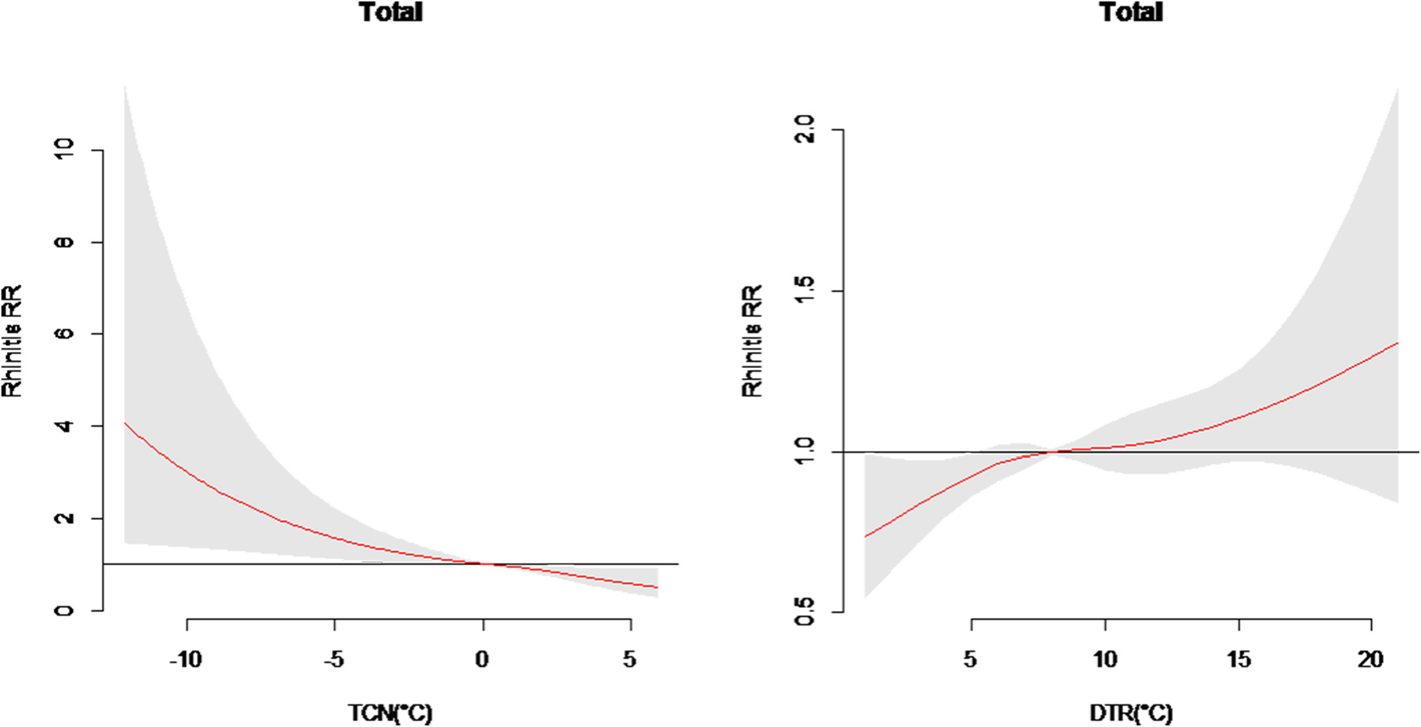
The overall effects of TCN and DTR on total childhood AR

Figure 3 shows the exposure-response relationships of TCN and DTR with childhood AR. It suggested that the temperature decrease between two adjacent days (TCN < 0 °C) was associated with increased risk of childhood AR, while DTR seemed not to have adverse effects on childhood AR.

**Fig. 4:**
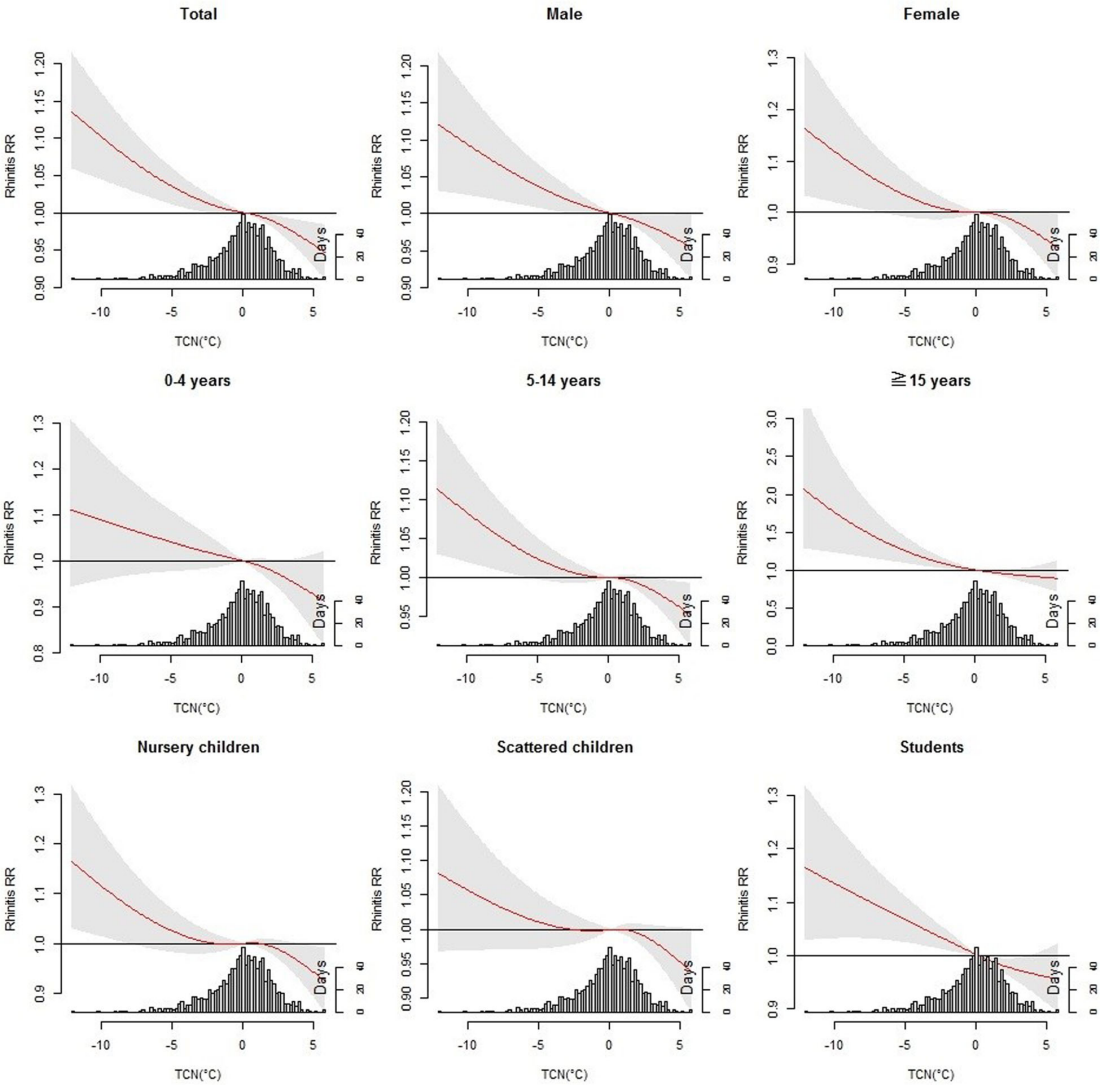
The overall effects of TCN on total childhood AR. of TCN on sex, age, and occupation

Figure 4 shows the association between childhood AR and TCN stratified by sex, age, and occupation at lag 12. Significant association was observed for both sex, children aged ≥5 years, as well as nursery children and students.

Sensitivity analyses were conducted by changing the *df* (6–8 per year) for time to control for long-term trend and seasonality. We also varied the *df* (3–5) for temperature, humidity. We found that the results changed a little (results not shown).

## Discussion

It has been well documented that temperature variability within a short period (e.g., DTR and TCN) affects human health among children [15–17]. We carried out a standard time-series study to examine the effects of DTR and TCN on childhood AR in Hefei, China. We found that temperature drops between 2 days (TCN < 0) increased the risk of childhood AR. This risk was greater among boys, children ≥15 years and students. However, significant effect of DTR was not observed.

The association between temperature and AR was examined in several recent studies, consistently showing an adverse effect of cold weather on AR in many countries such as Korea, China and Finland [12–14]. However, it still remains unknown whether or not the risk of AR could be influenced by unstable weather, i.e., DTR or TCN. In this contribution, we used a standard time-series analysis to explore the association between childhood AR and temperature variability. Relevant findings provide support for our hypothesis that there is a connection between TCN and AR in children. Similarly, some previous studies also reported adverse effect of short-term temperature variability. For example, Xu and colleagues found the risk of childhood asthma increased above a DTR of 10 °C [17, 18] reported a 1.0% increase of childhood acute bronchitis cases per 1 C increment of DTR. The present study further revealed that temperature variability had adverse effects on childhood AR.

Although DTR and TCN have been widely used in previous studies, litter is known about the relative effects of these two temperature variability indictors. To our knowledge, only one study has evaluated the health risk of DTR and TCN among children [18]. This study was conducted in Brisbane, Australia, which found significant association of childhood pneumonia with TCN, not DTR. The present study also suggested that the risk of AR among children was particularly vulnerable to the effects of TCN, rather than DTR. These findings probably imply that more attention paid to the temperature change between two adjacent days could help reduce the negative health consequences of unstable weather among children.

We also observed that TCN had the largest effect 1 week later, namely at lag 12 (Table 2). One possible reason for the observed delayed effects of TCN is that, after exposure to temperature variability, our thermoregulatory center and immune system will be involved to eliminate or alleviate the harmful health effects of temperature. Few days later, when temperature effects exceed the limit that our proactive defense can handle, some respiratory diseases could occur such as allergic rhinitis as well as childhood pneumonia as reported previously [18]. Another reason could be related to the delayed effects of TCN on the dehydration of pollen, which is the trigger of allergic rhinitis. Nevertheless, more research is needed to explore the mechanism for the delayed effects of temperature variability.

Stratified analyses by sex revealed that boys were more likely to be influenced by TCN than girls. In general, boys have more outdoor activities than girls, which may expose them more often to outdoor environment change such as temperature drops, increasing the possibility of AR infection. Stratified analyses by age suggested that children aged 15 year or above and in-school children (i.e., students) were more vulnerable to TCN. This result contradicts with our commonsense that, compared with old children, younger children are more vulnerable to temperature effects, because younger children are relatively under-developed and have less self-care ability [16]. Therefore, more research is needed to prove our findings in other regions.

This study has some strengths. To be best of our knowledge, this is the first study to date investigating the association of AR with short-term temperature variability. Our findings thus add new evidence to the existing literature of temperature and AR. In the preset study, temperature variability was measured by DTR and TCN; these two temperature indictors were often employed by previous researchers to evaluate the effect of unstable weather on human health [16, 19]. Our results regarding the comparison of the effects of DTR and TCN will help us to know that the major threat of temperature variability to AR occurrence is from sudden temperature drop between two adjacent days. We were also able to perform stratified analyses by age, sex, occupation; relevant findings will be useful for guiding the prevention of AR occurrence from the adverse effect of temperature. Limitations of this study should be also acknowledged. First, this study was conducted in a single city, which limits the generalization of our findings to other regions with distinct city characteristics such as climate and socioeconomic status. Second, similar to previous study, we used the mean temperature to measure the exposure of temperature among children, which to some extent caused some biases [16, 19]. Besides, many other confounders such as airborne pollen are risk factors of allergic rhinitis have not been considered in our data analysis. The effects of TCN may be attenuated after including more confounders in the data analysis. Third, this is an ecological study, which cannot prove the causal association between temperature variability and AR. Fourth, the type of allergic sensitization (pollen, mold, and mites) play an important role in influencing the association between environmental risk factors and allergic rhinitis. Equally important is the usage of medicine among patients with allergic rhinitis. Therefore, the association between temperature variability and allergic rhinitis could be modified by the type of allergic sensitization (pollen, mold, mites) as well as some medicines for treating allergic rhinitis. However, we were unable to conduct such stratified analyses in the present study because the relevant data were not available.

## Conclusions

In total, this study provides evidence that exposure to a large temperature drop between two adjacent days is associated with increased risk of AR in children. Vulnerable subgroups who are at higher risk of AR are boys and children older than 15 years. Temperature variability should be taken into account in future research assessing the deleterious effect of temperature on AR among children.

## Data Availability

Daily data on emergency room visits and outpatients for childhood allergic rhinitis are available from Anhui Provincial Children’s Hospital, China. All data used for analysis are available upon a proper request from the corresponding author Hong Ni at nhong666@163.com

## Abbreviations

AIC: Akaike Information Criterion
AR: allergic rhinitis
df: degrees of freedom
DLNM: distributed lag non-linear model
DTR: diurnal temperature range
ICD: International Classification of Diseases
QOL: quality of life
RR: relative risk
TCN: temperature change between two neighboring days
